# Fruit Ripening Regulation of α-Mannosidase Expression by the MADS Box Transcription Factor RIPENING INHIBITOR and Ethylene

**DOI:** 10.3389/fpls.2016.00010

**Published:** 2016-01-21

**Authors:** Mohammad Irfan, Sumit Ghosh, Vijaykumar S. Meli, Anil Kumar, Vinay Kumar, Niranjan Chakraborty, Subhra Chakraborty, Asis Datta

**Affiliations:** ^1^National Institute of Plant Genome Research New Delhi, India; ^2^Central Institute of Medicinal and Aromatic Plants, Council of Scientific and Industrial ResearchLucknow, India; ^3^Department of Biological Chemistry, David Geffen School of Medicine, University of California, Los Angeles, Los AngelesCA, USA

**Keywords:** fruit ripening, transcriptional regulation, fruit ripening-specific promoter, α-Man, RIN

## Abstract

α-Mannosidase (α-Man), a fruit ripening-specific *N*-glycan processing enzyme, is involved in ripening-associated fruit softening process. However, the regulation of fruit-ripening specific expression of *α-Man* is not well understood. We have identified and functionally characterized the promoter of tomato (*Solanum lycopersicum*) *α-Man* to provide molecular insights into its transcriptional regulation during fruit ripening. Fruit ripening-specific activation of the *α-Man* promoter was revealed by analysing promoter driven expression of *beta-glucuronidase* (*GUS*) reporter in transgenic tomato. We found that RIPENING INHIBITOR (RIN), a MADS box family transcription factor acts as positive transcriptional regulator of *α-Man* during fruit ripening. RIN directly bound to the *α-Man* promoter sequence and promoter activation/α-Man expression was compromised in *rin* mutant fruit. Deletion analysis revealed that a promoter fragment (567 bp upstream of translational start site) that contained three CArG boxes (binding sites for RIN) was sufficient to drive *GUS* expression in fruits. In addition, *α-Man* expression was down-regulated in fruits of *Nr* mutant which is impaired in ethylene perception and promoter activation/*α-Man* expression was induced in wild type following treatment with a precursor of ethylene biosynthesis, 1-aminocyclopropane-1-carboxylic acid (ACC). Although, *α-Man* expression was induced in *rin* mutant after ACC treatment, the transcript level was less as compared to ACC-treated wild type. Taken together, these results suggest RIN-mediated direct transcriptional regulation of *α-Man* during fruit ripening and ethylene may acts in RIN-dependent and -independent ways to regulate *α-Man* expression.

## Introduction

Tomato (*Solanum lycopersicum*) is an important component of human diet and also serves a model for biochemical and genetic analysis of fleshy fruit development and ripening process. The ripening of fleshy fruits is accompanied by a number of biochemical events, including changes in color, sugar, acidity, texture, and aroma volatiles that are crucial for the development of sensory qualities of fruits (Klee and Giovannoni, 2011; Osorio et al., 2012; Seymour et al., 2013a,b). During ripening, fruit textural changes are brought about by concerted and coordinated activities of the enzymes that act upon cell wall components such as cellulose, hemicellulose, pectin and *N*-glycoproteins (Giovannoni, 2001; Brummell, 2006; Meli et al., 2010; Klee and Giovannoni, 2011). An earlier report suggested that blocking of *N*-glycosylation can lead to delay of fruit ripening (Handa et al., 1985). Moreover, during ripening, tomato pericarp has been shown to accumulate high amount of free *N*-glycans as precursors of *N*-glycosylation or as a result of *N*-glycoprotein degradation (Priem et al., 1993; Nakamura et al., 2008; Hossain et al., 2009). These free *N*-glycans can also stimulate fruit ripening by inducing ethylene biosynthesis and signaling (Priem and Gross, 1992). A more mechanistic insight on the role of *N*-glycans in fruit ripening was obtained by studying two *N*-glycan processing enzymes, α-mannosidase (α-Man) and β-D-*N*-acetylhexosaminidase (β-Hex). The suppression of expression of these enzymes during tomato ripening had resulted in enhancement of fruit shelf life due to reduced softening of fruits during ripening (Meli et al., 2010; Ghosh et al., 2011; Cao et al., 2014). Although, regulation of β-Hex expression during fruit ripening was being studied in detail (Irfan et al., 2014); our knowledge of how α-Man expression is regulated during fruit ripening is still not clear.

α-Man (EC 3.2.1.24) is a member of the glycosyl hydrolase 38 (GH38) family carbohydrate acting enzymes reported in animals, plants and microorganisms. It cleaves terminal α-mannosidic linkages from both the high mannose type and complex type *N*-glycans present in glycoproteins (Strasser et al., 2006; Hossain et al., 2009; Liebminger et al., 2009; Hüttner et al., 2014). α-Man activity was shown to increase during ripening of several fleshy fruits such as tomato, capsicum, mango, papaya (Priya Sethu and Prabha, 1997; Suvarnalatha and Prabha, 1999; Hossain et al., 2009; Meli et al., 2010; Ghosh et al., 2011). The genes encoding fruit ripening-specific α-Man have been identified and characterized from tomato and capsicum (Hossain et al., 2010; Meli et al., 2010; Ghosh et al., 2011). Functional characterization of *α-Man* in climacteric fruit tomato and non-climacteric fruit capsicum revealed its involvement in ripening-associated fruit softening (Meli et al., 2010; Ghosh et al., 2011). Interestingly, the genes encoding enzymes involved in cell wall loosening, e.g., pectin methyl esterase, glucan endo1,3-β-D-glucosidase, β 1,3 glucanse, endo-xyloglucan transferase, pectin esterase, expansions, pectin acetyl esterase, α-galactosidase, pectate lyase, (1-4)-β-mannan endohydrolase and β-galactosidase were down-regulated in *α-Man*-RNAi tomato fruits (Meli et al., 2010). Moreover, the expression of ethylene biosynthesis genes (ACC synthase, and ACC oxidase) and transcription factors like ERFs were also down-regulated in *α-Man*-suppressed tomato fruits, suggesting that α-Man-mediated cleavage of terminal mannose residues attached to the cell wall *N*-glycoproteins may be involved in positive feed-back regulation of fruit ripening (Meli et al., 2010).

The MADS box family transcription factor, RIN (RIPENING INHIBITOR) plays a pivotal role in fruit ripening by controlling the transcription of a large number of genes involved in cellular signaling and metabolism (Giovannoni, 2007; Ito et al., 2008; Fujisawa et al., 2011, 2013; Zhong et al., 2013; Kumar et al., 2015). Tomato *rin* mutant fruit was characterized by enlarged sepals and completely inhibited ripening process, due to the abnormal expression of two genes encoding the RIN and MC (MACROCALYX). Although RIN regulates fruit ripening, MC is involved in sepal development (Vrebalov et al., 2002). RIN-dependent transcriptional regulation of fruit ripening has been well studied following chromatin immunoprecipitation, transcriptome and proteome analyses. This led to identification of several direct and indirect targets of RIN including those involved in biosynthesis and signaling of phytohormone ethylene, cell wall modification, and accumulation of carotenoid and aroma volatiles. (Ito et al., 2008; Fujisawa et al., 2011, 2012, 2013; Martel et al., 2011; Kumar et al., 2012, 2015; Qin et al., 2012; Zhong et al., 2013). Besides, the expression of some key transcriptional regulators namely NON-RIPENING, COLORLESS NON-RIPENING, FRUITFULL1 was also found to be under the transcriptional control of RIN (Fujisawa et al., 2011, 2012, 2013, 2014; Martel et al., 2011).

A strong correlation between *α-Man* transcript and protein accumulation patterns in tomato fruits (Meli et al., 2010), suggested that the fruit ripening-specific activity of α-Man is under the transcriptional control of gene expression. Therefore, to gain an insight into transcriptional regulation of *α-Man* during fruit ripening, the promoter of tomato *α-Man* was isolated and functionally characterized. The activation of *α-Man* promoter during fruit development and ripening was studied by analyzing promoter-driven expression of *beta-glucuronidase* (*GUS*) reporter in transgenic tomato plants. The results suggest that ethylene and RIN play important role in controlling the transcription of *α-Man* during fruit ripening.

## Materials and Methods

### Plant Materials and Growth Conditions

Seeds of *Solanum lycopersicum* cv. Pusa Ruby were collected from the National Seeds Corporation Ltd, New Delhi and *Solanum lycopersicum* cv. Ailsa Craig, *rin* and *Nr* mutants were obtained from Tomato Genetics Resource Center, University of California at Davis. Seeds were germinated in small pots and after 3 weeks, seedlings were transplanted to big pots in the greenhouse conditions; 25/25°C temperature, 70% humidity under 14/10 h light/dark regime. Flowers were tagged at anthesis and fruits from various developmental stages [3, 5, 10, 15, and 20 days after anthesis (DAA)] and ripening stages [mature green (MG), breaker (BR), pink (P), and red ripe (RR)] were harvested for various type analysis of fruits.

### Isolation and *In Silico* Analysis of *α-Man* Promoter

The Universal GenomeWalkerTM Kit (Clontech, USA) was used for isolation of *α-Man* promoter from tomato. Genomic DNA was extracted from leaves by using cetyl trimethyl ammonium bromide (CTAB) method described by Doyle and Doyle (1987). Five genome walking libraries were prepared by digesting genomic DNA separately with *Pvu*II, *Xmn*I, *Msc*I, *Dra*I, and *Ssp*I enzymes. After that, PCR was carried out with a GenomeWalker adapter-specific primer (AP1) and a gene-specific primer (GSP1) separately for each library. This PCR product was then used as a template for nested amplification by using AP2 and GSP2 primers followed by cloning in pGEM-T Easy vector and then sequencing the clones. The promoter sequence thus obtained was further verified with sequence available on www.solgenomics.net. Tomato *α-Man* promoter sequence was analyzed *in silico* to find out putative *cis*-acting elements using NewPLACE (Higo et al., 1999), PlantCARE (Lescot et al., 2002), and MatInspector (Cartharius et al., 2005) servers, and the FUZZNUC program (EMBOSS package; Rice et al., 2000).

### Construction of Promoter::GUS Fusion and Deletion Vectors

Tomato *α-Man* promoter::GUS fusion construct (MP::GUS) and deletion constructs of α-Man promoter were prepared in binary vector pBI121 by replacing CaMV 35S promoter with α-Man promoter. Tomato full length *α-Man* promoter (1155 bp) and deletion fragments of α-Man promoter (-779 bp, -567 bp, -373 bp, and -187 bp region from translational start site of α-Man) were PCR amplified using high fidelity *pfx* DNA polymerase to incorporate appropriate restriction sites. Further, these amplified regions were cloned upstream to ATG of GUS gene in pBI121 binary vector following standards restriction digestion and ligation methods. All these constructs were named as MP::GUS (with 1155 bp long promoter), MD1::GUS (with (779 bp fragment of *α-Man* promoter), MD2::GUS (567 bp fragment of *α-Man* promoter), MD3::GUS (373 bp of *α-Man* promoter) and MD4::GUS (187 bp of *α-Man* promoter) respectively. The positive clones were transformed into *Agrobacterium tumefaciens* (strain EHA105) following electroporation. Transformation in *Agrobacterium* was confirmed by colony PCR using all these plasmid DNA as template and set of primers enlisted in **Supplementary Table S2**.

### *Agrobacterium*-Based Transient Assay

*Agrobacterium*-based transient assay was carried out as described previously (Orzaez et al., 2006) with few modifications. In the pericarp of tomato fruits (*Solanum lycopersicum* cv. Pusa Ruby) of mature green stage, *Agrobacterium* suspension was infiltrated. In brief, *Agrobacterium* cultures (3 mL) were grown overnight from individual colonies (transformed with appropriate construct) at 28°C in YEP medium plus selective antibiotics. Two hundred micro liter of it was transferred to 50 mL induction medium (0.5% beef extract, 0.1% yeast extract, 0.5% Peptone, 0.5% Sucrose, 2 mM MgSO_4_, 20 mM acetosyringone, 10 mM MES, pH 5.6) plus antibiotics, and again grown overnight until the OD_600_ of the culture reached 0.8–1.0. After centrifugation, cultures were recovered, resuspended in infiltration medium (10 mM MgCl_2_, 10 mM MES, 200 mM acetosyringone, pH 5.6) and incubated at room temperature with gentle agitation (20 rpm) for at least 2 h and then infiltrated into the fruits.

### Stable Transformation of Tomato

The transgenic tomato plants were developed as described previously (Fillati et al., 1987) with few modifications. Initially, seeds were sterilized using 4% commercial bleach and kept on Murashige and Skoog (MS) medium for germination. Cotyledons from 2-week-old seedlings were cut and co-cultivated with *Agrobacterium* transformed with appropriate construct in MS medium containing acetosyringone (0.1 μM). Cotyledons were dried and then kept for selection on MS plates containing kanamycin (50 mgl^-1^), cefotaxime (250 mgl^-1^) and zeatine (1 ngl^-1^). After few days, regenerated plantlets were transferred to rooting medium [MS containing kanamycin (50 mgl^-1^), cefotaxime (250 mgl^-1^) and IAA (1 ngl^-1^)]. Transgenic seeds were collected and then germinated in MS medium containing kanamycin (50 mgl^-1^) to get the progeny plants.

### RNA Isolation and qRT-PCR

RNA was extracted as protocol described previously (Menke et al., 1999) and purified using the RNeasy Mini Kit (Qiagen). Quantification of total RNA was carried out using a nanodrop (ND 1000) and five micrograms of RNA was reverse transcribed to cDNA using superscript II RT (Invitrogen). qRT-PCR analysis was performed using One Step Real Time RT PCR (Applied Biosystems) with SYBR Green as described by previously (Ghosh et al., 2013). All the qRT-PCR analysis was performed in triplicate from cDNA derived from at least two independent experiments. The data was analyzed by using the 2^-ΔΔCT^ method (Bovy et al., 2002) and presented as fold change in gene expression or percentage of expression, normalized to the endogenous control (tomato actin gene). The primers used for qRT-PCR reaction are listed in **Supplementary Table S2**.

### GUS Histochemical and Fluorometric Assay

Detailed histochemical analysis of the reporter gene (GUS) was carried out as method described by Jefferson et al. (1987) with few modifications. The transverse sections of fruits from different development and ripening stages, seedlings, roots, leaves, and flowers were dipped in GUS staining solution (100 mM sodium phosphate buffer, pH 7.0, 10 mM EDTA, 0.5 mM K_3_Fe(CN)_6_, 0.5 mM K_4_Fe(CN)_6_, 0.1% triton-X 100, 20% methanol and 1 mM X-gluc). After vacuum infiltration, the plant materials were left for overnight at 37°C in darkness. To remove chlorophyll, samples were destained in 75% ethanol and then photographs were taken by using Canon G6 powershot with 4X zoom or Canon EOS 400D DIGITAL (10.1 megapixel) and Nikon AZ100 5X microscope. Further, GUS activity was quantified by measuring the production of 4-methylumbelliferone (4-MU) as pmol 4-MU mg^-1^min^-1^. To do this, samples were homogenized in 400 μl GUS extraction buffer (50 mM sodium phosphate buffer, pH 7.0, 10 mM DTT, 10 mM EDTA, 0.1% sodium lauryl sarcosine, 0.1% triton-X 100) and then centrifuged. After that, 50 μl of supernatant was mixed to 450 μl MUG assay buffer (GUS extraction buffer containing 10 mM MUG) and incubated at 37°C for 1 h. The reaction was terminated by mixing 100 μl of aliquots with 900 μl 0.2 M Na_2_CO_3_. The fluorescence was recorded in fluorometer (Cary Eclipse, Varian) with excitation at 380 nm and emission at 454 nm. GUS activity was calculated as pmoles MU mg protein^-1^min^-1^.

### Electrophoretic Mobility Shift Assay

EMSA was performed for *in vitro* binding of RIN (AF448522.1) protein with *α-Man* promoter as methods described previously (Irfan et al., 2014) with minor modifications. Briefly, 167 bp long fragment of *α-Man* promoter upstream of ATG of gene was PCR amplified by using primers listed in **Supplementary Table S1**. *Hind*III/*Xba*I digested α-Man promoter fragment was end filled with [α-P^32^]dCTP (3000 Ci/mmol, 50 μCi), using DNA polymerase I (Klenow) fragment (New England Biolabs) and purified using sephadex G-50 column. EMSA was performed with [α-P^32^]dCTP labeled α-Man promoter fragment incubated with RIN protein purified by Irfan et al. (2014) in gel shift assay binding buffer (20 mM HEPES, pH 7.5, 20% glycerol, 0.05 μg poly(dIdC):poly(dIdC), 10 mM MgCl_2_, 2.5 mM EDTA, 2.5 mM DTT and 25 mM NaCl) at 25°C for 30 min. For competition assay, unlabeled promoter fragment was used as specific competitive inhibitor and an unrelated DNA [200 bp region downstream of ATG of tomato actin (FJ532351.1)] was used as non-specific competitor. After incubation for 30 min, reaction was loaded onto 6% native PAGE. The gel was run at room temperature at constant current of 10 mA using 0.5X TBE running buffer. The protein-DNA complexes as well as free probes were visualized by autoradiography.

### ACC Treatment

Three weeks old tomato seedlings, germinated on MS media were transferred to liquid MS media containing 1 mM ACC. The tissue was harvested at different time points and frozen immediately in liquid nitrogen. Seedlings transferred to MS liquid media without ACC were used as control.

## Results

### Isolation and *In Silico* Analysis of *α-Man* Promoter

**FIGURE 1:**
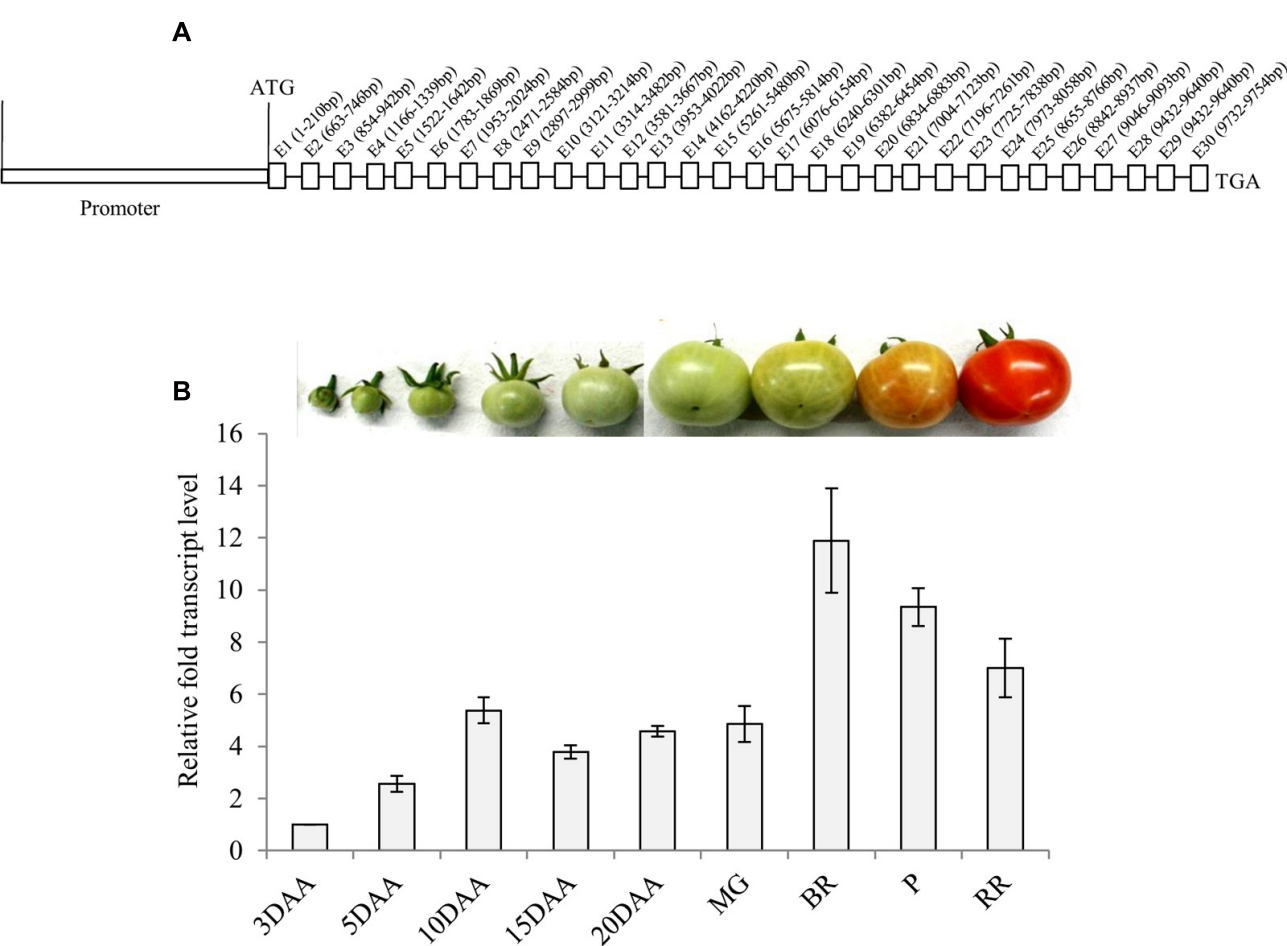
***α-Man* genomic organization and transcript abundance pattern during tomato fruit development and ripening.** **(A)** Genomic organization of *α-Man*. Introns on *α-Man* gene were identified following intron finder tool (solgenomics.net). **(B)** Expression analysis of *α-Man* during fruit development and ripening. RNA was isolated from fruits of various developmental stages (3DAA–20DAA) and ripening stages (MG, BR, P and RR). The transcript level of *α-Man* was determined by qRT-PCR analysis. Tomato actin was used as endogenous control. Data are mean (±SE) of three biological replicates.

Analysis of the tomato genomic sequence^1^ revealed that *α-Man* gene (Solyc06g068860.2.1) contains 30 exons interrupted by 29 introns (**Figure 1A**). The upstream sequence of tomato *α-Man* was initially isolated by directional genome walking PCR using a set of adapter and gene-specific primers based on the cDNA sequence information (EU244853) (**Supplementary Figure S1**). The sequence (1155 bp) upstream of the translational start site, obtained from the genome walking PCR, was verified with genomic sequence of tomato *α-Man* available on solgenomics network database^1^. In order to identify putative *cis*-acting regulatory elements that might control the transcription of *α-Man*, *in silico* analysis of upstream sequence, i.e., putative promoter sequence, was carried out by using NewPLACE^2^, PlantCARE (bioinformatics.psb.ugent.be), and MatInspector^3^. In addition to the ubiquitous elements including TATA, CAAT boxes, the promoter region of tomato *α-Man* contains sequences similar to the *cis*-acting regulatory elements found within the promoter of other plant genes (**Supplementary Table S1**). The promoter region of *α-Man* contains several putative functional *cis*-acting elements recognized by the transcription factors, which may be involved in perceiving stimulus from different plant hormones and environmental stresses (**Supplementary Table S1**). Interestingly, *in silico* analysis also revealed the presence of RIN binding sites (CArG boxes) within the *α-Man* promoter (**Supplementary Table S1**) which suggested the possible role of RIN in the regulation of *α-Man* transcript expression during fruit ripening.

### Histochemical, Fluorometric and Transcript Analysis of *α-Man* Promoter::*GUS* Transgenic Lines

**FIGURE 2:**
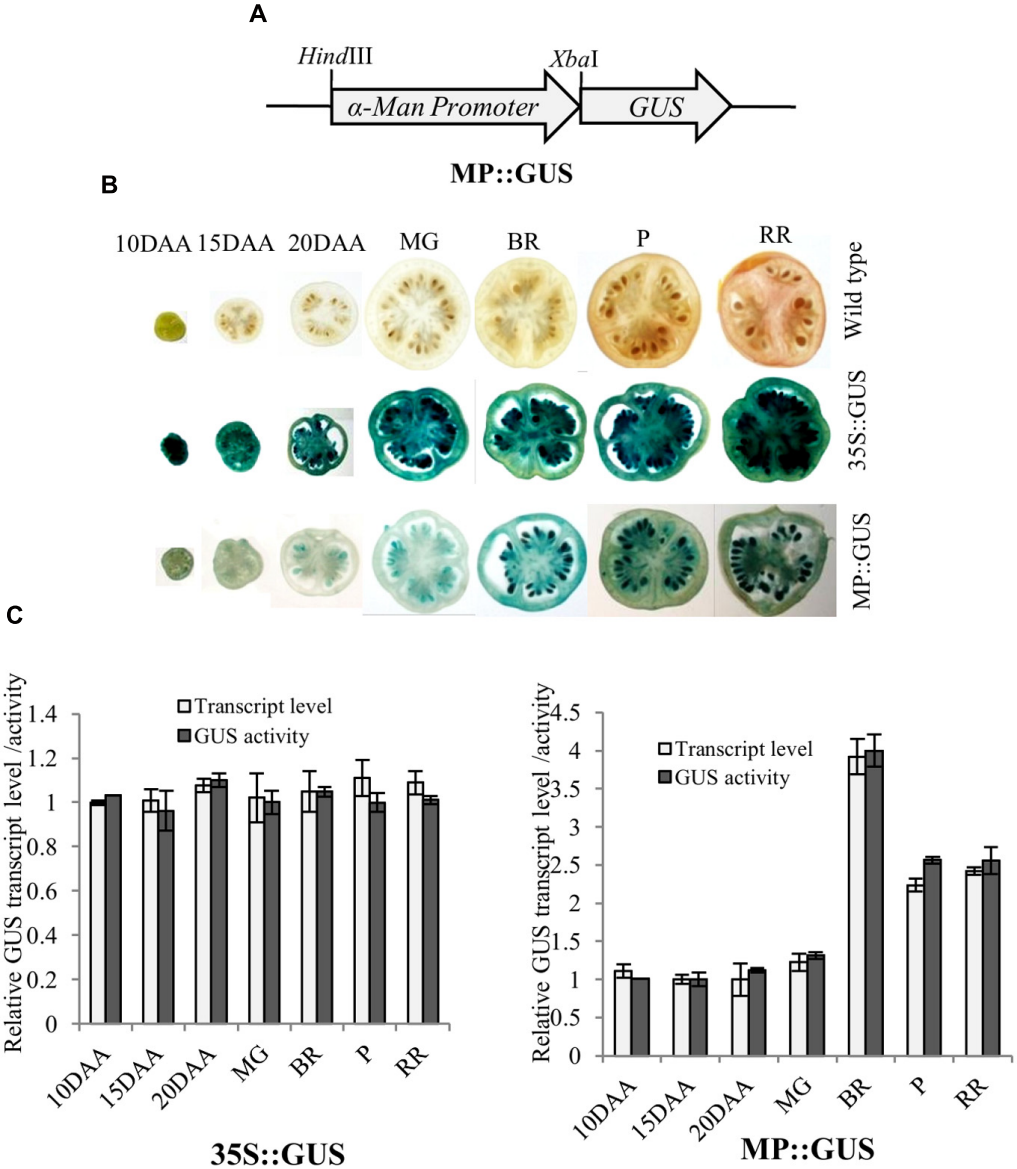
**Histochemical and fluorometric assays of GUS activity in fruits.** **(A)** Schematic representation of MP::GUS fusion construct (*GUS* fused with *α-Man* promoter). **(B)** Histochemical GUS staining was carried out in fruits of different developmental and ripening stages. MP::GUS and 35S::GUS denote MP and CaMV35S promoter driven expression of *GU*S. Wild type was used as untranformed control. **(C)** Accumulation of *GUS* transcript and activity in transgenic fruits were measured by qRT-PCR and fluorometric GUS assay, respectively. Tomato actin was used as endogenous control in qRT-PCR. Data are mean (±SE) of three biological replicates.

**FIGURE 3:**
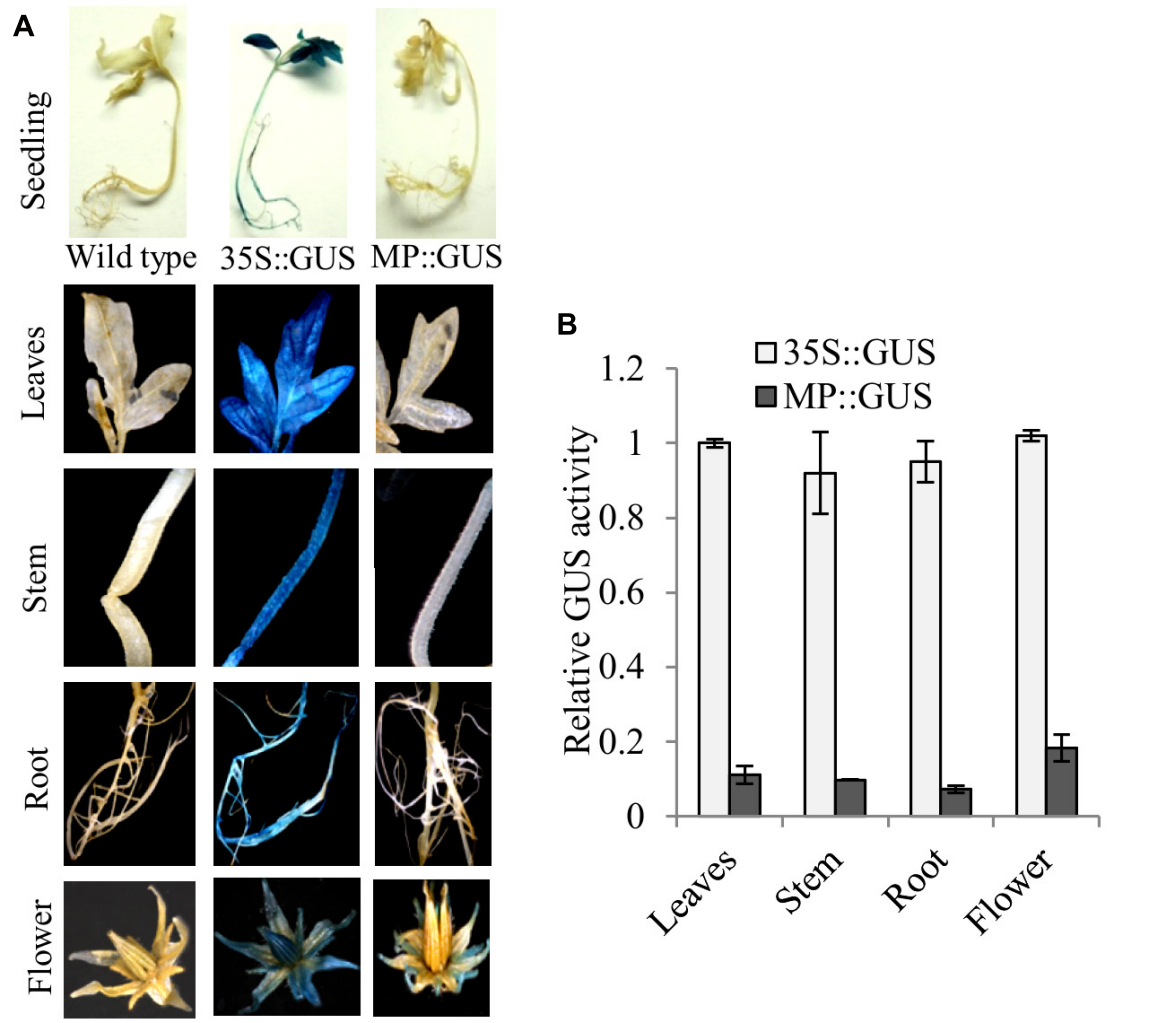
**Histochemical and fluorometric assays to detect GUS activity in different plant parts.** **(A)** Seedling, leaves, stem, root, and flower from MP::GUS, 35S::GUS and wild type (untransformed) were subjected to histochemical staining. **(B)** Fluorometric analysis of GUS activity. Data are presented as the mean (±SE) of three biological replicates.

Fruit-specific expression of *α-Man* during tomato ripening, with maximum transcript expression at the breaker stage, was reported earlier following northern blot analysis (Meli et al., 2010). To validate the expression pattern of *α-Man* during fruit ripening, transcript level was quantified by qRT-PCR during tomato fruit development and ripening stages. The results of qRT-PCR analysis corroborated earlier findings that *α-Man* transcript accumulates during ripening with maximum level at breaker stage (**Figure 1B**). However, during fruit developmental stages (3DAA, 5DAA, 10DAA, 15DAA, 20DAA) a basal level expression of *α-Man* was noticed (**Figure 1B**). In order to gain further insight into fruit ripening-specific activation, *α-Man* promoter driven expression of the *GUS* reporter was studied in transgenic tomato plants. *α-Man* promoter (-1 to -1155 bp upstream of ATG) was cloned into pBI121 binary vector in order to make MP::GUS fusion construct (**Figure 2A**). Fifteen independent transgenic events, developed with MP::*GUS* fusion construct were advanced for T2 generation and confirmed by PCR (**Supplementary Figure S2**). T2 transgenic events with single transgene copy insertion were chosen for further analysis. To examine the tissue-specific expression pattern, fruits at different development [10, 15, and 20 days after (DAA)] and ripening (mature green, breaker, pink, and red ripe) stages, leaves, roots, and flowers from transgenic plants were subjected to histochemical GUS staining as described in Material and methods (**Figures 2B** and **3A**). Intense GUS staining of fruits during ripening with a peak at breaker stage was observed when *GUS* expression was driven by *α-Man* promoter in transgenic fruits (**Figure 2B**). GUS activity was apparently not detectable by visual observations of seedlings, leaves, stems and roots of transgenic plants transformed with MP::GUS fusion construct (**Figure 3A**). The flowers of transgenic plants harboring *α-Man* promoter showed very less GUS staining mainly in sepals, while there was no GUS staining in petals (**Figure 3A**). The results obtained from histochemical GUS staining were further validated by quantifying GUS activity through fluorometric MUG (4-methylumbelliferone glucuronide) assay. In *α-Man* promoter transgenic fruits, GUS activity was higher at breaker stage of fruit ripening as compared with other stages (**Figure 2C**). The CaMV 35S::GUS transgenic plants showed constitutive GUS activity in fruits, seedlings, leaves, stem, roots, and flowers (**Figures 2B,C** and **3A,B**) whereas wild type (cv. Pusa Ruby) plants did not show GUS activity in any parts of the plant (**Figure 2B**). To further corroborate these results, *GUS* transcript accumulation pattern was studied following qRT-PCR. Maximum level of *GUS* mRNA accumulated during fruit ripening stages which was in accordance with GUS histochemical and fluorometric results (**Figure 2C**). Therefore, 1155 bp sequence upstream sequence from ATG can be regarded as the full-length *α-Man* promoter. The promoter sequence contains *cis*-acting elements that may be involved in fruit-ripening specific expression of *α-Man* (**Supplementary Table S1**) and the promoter was able to drive tissue-specific expression of *GUS* similar to that observed for the endogenous expression of *α-Man* (**Figures 1B** and **2B,C**).

### Deletion Analysis of *α-Man* Promoter

**FIGURE 4:**
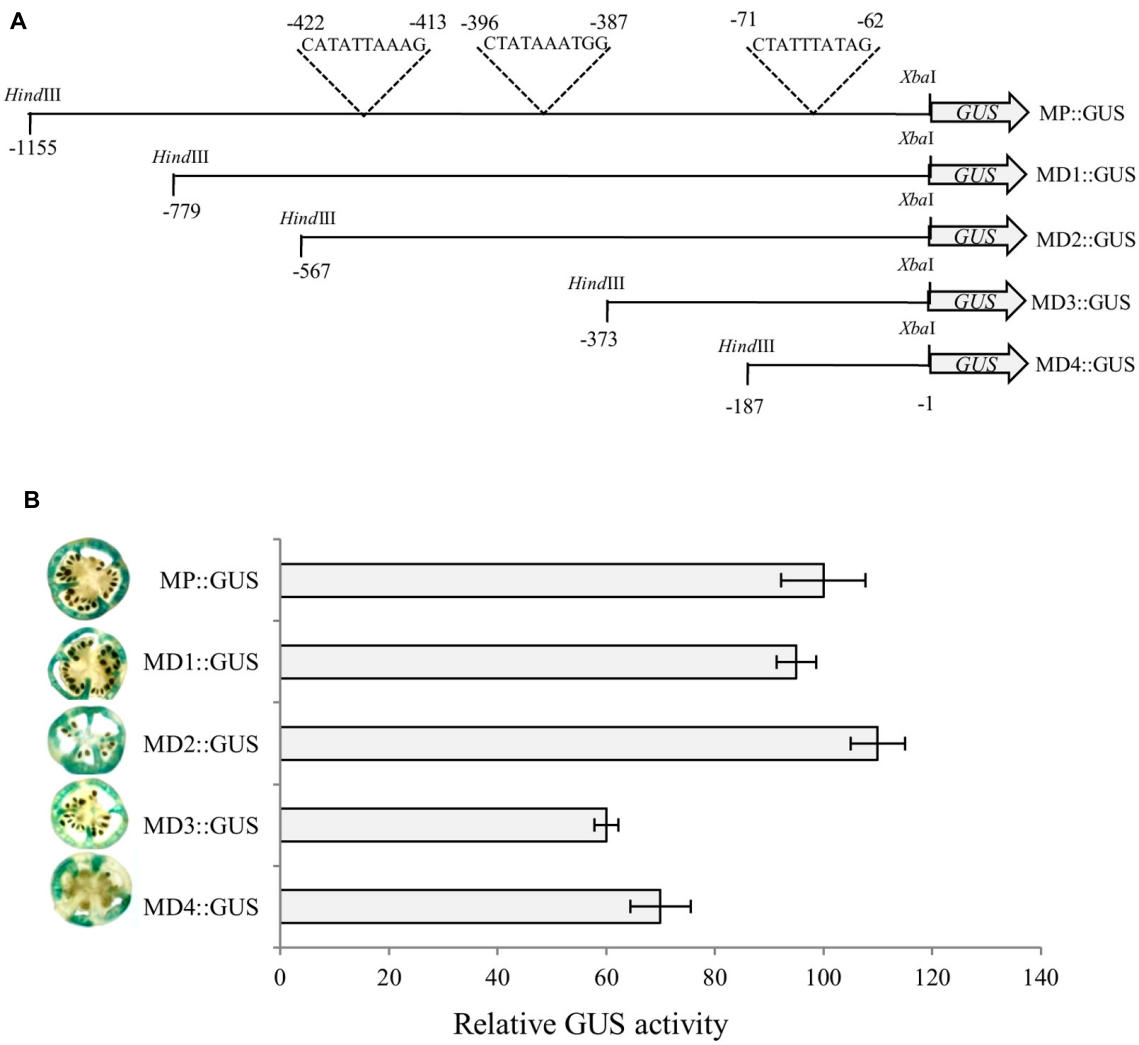
**Deletion analysis of *α-Man* promoter.** **(A)** Schematic representation of promoter deletion constructs. 187, 373, 567, and 779 bp regions upstream of translational start site were cloned into pBI121 binary vector in place of CaMV35S promoter to generate *GUS* fusion constructs (MD4::GUS, MD3::GUS, MD2::GUS and MD1::GUS respectively). **(B)** Analysis of promoter driven expression of *GUS*. Histochemical and fluorometric GUS assays were carried out in fruits after *Agrobacterium*-mediated transient expression. Data are presented as the mean (±SE) from three biological replicates.

To identify optimal promoter region required for *α-Man* expression in fruit, deletion analysis of the promoter was carried out. Four deletion constructs (MD1::GUS, MD2::GUS, MD3::GUS, and MD4::GUS) that included 779, 567, 373 and 187 bp upstream of ATG, respectively, were designed to study *GUS* reporter expression pattern, controlled by the *α-Man* promoter fragments (**Figure 4A**, **Supplementary Figures S3** and **S4**). Functional analysis of these promoter::*GUS* constructs was carried out by *Agrobacterium*-mediated transient expression (Agroinjection) of GUS gene in tomato fruits as described in “Materials and Methods.” Fruits were harvested 3 days after agroinjection in order to assess the time period necessary for measuring reporter expression and cut into sections and analyzed for GUS activity by histochemical assay and fluorometric MUG assay. GUS expression in fruits driven by MD3 and MD4 fragment of *α-Man* promoter was significantly less as compare to the full length and other promoter fragments (**Figure 4B**). These results were further validated by fluorometric quantification of GUS activity by MUG assay. The MUG assay also demonstrated that *α-Man* promoter activity was affected in MD3::GUS and MD4::GUS injected tomato fruits (**Figure 4B**). *In silico* analysis of these promoter fragments revealed the presence of RIN binding sites [CArG box, C(T/A/C)(AT)6(A/T/G)G] on these region (**Supplementary Figure S4**; **Supplementary Table S1**).

### Transcriptional Regulation of *α-Man* by RIN

**FIGURE 5:**
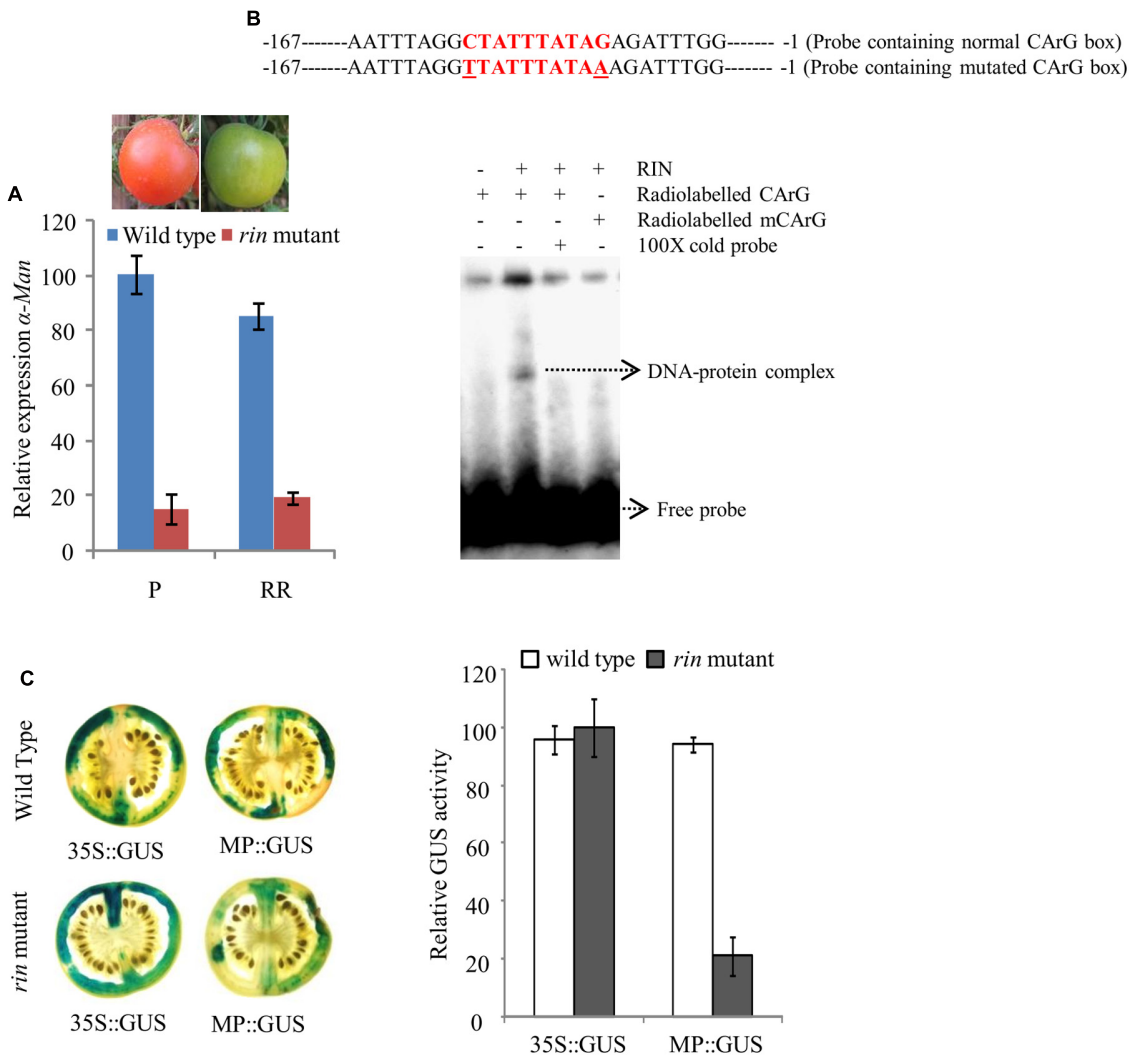
**RIN regulated expression of *α-Man* in fruit.** **(A)** Relative transcript abundance of *α-Man* in wild type (cv. Ailsa Craig) and *rin* mutant fruits was determined by qRT-PCR using tomato actin as endogenous control. **(B)** EMSA showing *in vitro* binding of RIN protein with the *α-Man* promoter. **(C)** CaMV35S and *α-Man* promoter driven expression of *GUS* in fruits of wild type and *rin* mutant. GUS activity in agroinjected fruits was analyzed by histochemical and fluorometric assays. Data are presented as the mean (±SE) of two biological replicates.

The qRT-PCR analysis of *α-Man* transcript level in wild type and *rin* mutant fruit revealed about 90% suppression of transcript level in *rin* mutant fruits (**Figure 5A**; Meli et al., 2010). Moreover, *in silico* analysis of *α-Man* promoter identified three CArG boxes (**Figure 4A**, **Supplementary Table S2**). CArG boxes are required for the direct binding of RIN to the promoter of ripening-specific genes (Ito et al., 2008; Fujisawa et al., 2011, 2013). In order to understand the role of RIN in regulation of *α-Man* expression, direct interaction of RIN with the *α-Man* promoter was tested by EMSA. For this a 187 bp fragment of promoter (MD4) that contained a CArG box closest to ATG, was used (**Figure 5B**, **Supplementary Figure S3**). Recombinant RIN protein which is known to bind to promoter of fruit ripening specific gene of β-Hex was purified from *Escherichia coli* cells and used for the EMSA (Irfan et al., 2014). When radiolabeled probe was incubated with recombinant RIN protein a shift was detected, whereas shift was not observed when the terminal C and G of CArG box was replace to T and A, respectively (**Figure 5B**). Moreover, signal was not detected when we included cold competitor in binding reaction (**Figure 5B**). Taken together these results suggest that RIN specifically binds to the CArG box element of *α-Man* promoter.

In order to further examine *α-Man* transcriptional regulation by RIN, *in fruto* transient expression assay of MP::GUS construct in *rin* mutant fruits was performed through Agroinjection. After histochemical staining, we observed that GUS activity in *rin* mutant fruit was significantly reduced as compared to wild type fruits, when GUS expression was driven by the *α-Man* promoter (**Figure 5C**). However, GUS activity was found to be almost similar in wild type and *rin* mutant fruits, when *GUS* expression was under the control of constitutive promoter. Fluorometric quantification assay also demonstrated a decrease in reporter gene activity conferred by *α-Man* promoter in *rin* mutant fruits as compared to wild type (**Figure 5C**). These results suggest that the transcription factor RIN positively regulates *α-Man* expression during tomato ripening.

### Regulation of *α-Man* by Ethylene

**FIGURE 6:**
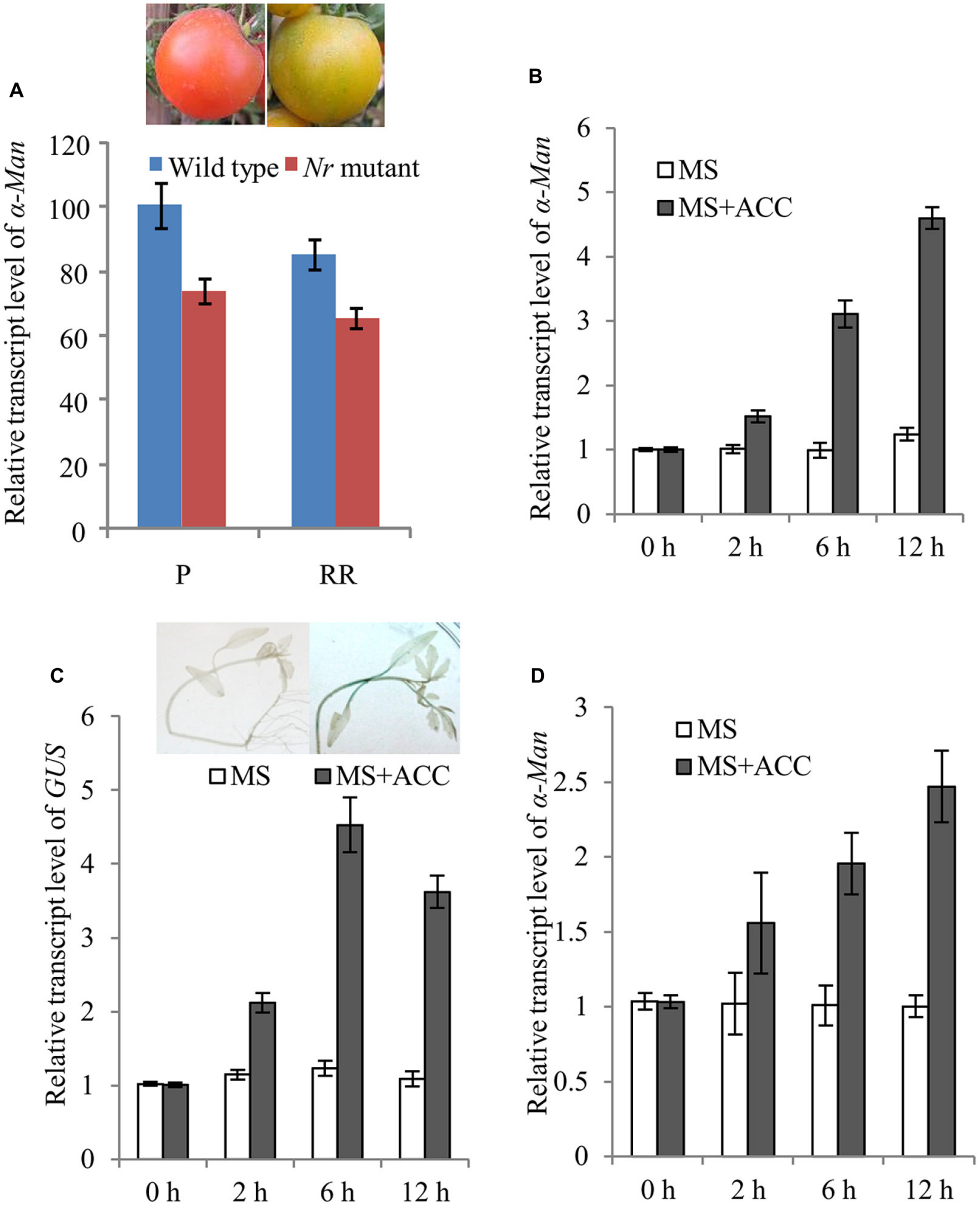
**Ethylene regulated expression of *α-Man*.** **(A)** qRT PCR expression analysis of *α-Man* in fruits of wild type (Ailsa Craig) and ethylene receptor *Nr* mutant. **(B)** Ethylene inducibility of *α-Man* was confirmed with qRT-PCR analysis of *α-Man* transcript level in wild type seedlings and **(C)** *GUS* transcript level in MP::GUS transgenic seedlings. Histochemical assay was carried out after 4 h of treatment. Data are presented as the mean (±SE) of three biological replicates. **(D)** Ethylene inducible expression of *α-Man* in *rin* mutant seedlings was determined by qRT-PCR analysis after ACC treatment.

Ethylene plays a vital role in ripening of climacteric fruits including tomato (Zegzouti et al., 1999; Giovannoni, 2001; Pech et al., 2012). Therefore, the role of ethylene in the regulation of *α-Man* expression was examined. The expression of *α-Man* in *Nr* mutant fruits was determined by qRT PCR. In *Nr* mutant, ripening is abolished due to mutation in NEVERRIPE (NR) receptor which percepts ethylene (Wilkinson et al., 1995). The transcript level of *α-Man* gene was decreased about 30% at the pink (P) and red ripe (RR) stages of *Nr* mutant fruit as compare to wild type fruit (**Figure 6A**). Through *in silico* analysis of *α-Man* promoter, ethylene responsive *cis*-acting elements and ethylene insensitive 3 (EIN3) like factors binding sites were also identified (**Supplementary Table S1**). To further understand the regulation of *α-Man* by ethylene, tomato seedlings were treated by ACC, the precursor of ethylene. ACC treatment to the wild type resulted in induced expression of *α-Man* (**Figure 6B**). Moreover, to check whether the promoter of *α-Man* was activated by ACC treatment, the expression of *GUS* was determined by qRT-PCR in MP::GUS transgenic seedlings after ACC treatment. The result revealed induced expression of *GUS*, suggesting activation of *α-Man* promoter after ACC treatment (**Figure 6C**). Although, RIN directly regulates the expression of ethylene biosynthesis and signaling genes, the expression of RIN is also under the control of ethylene (Lincoln and Fischer, 1988; Fujisawa et al., 2011, 2013; Martel et al., 2011; Osorio et al., 2011; Su et al., 2015). Therefore, we tested whether ACC treatment can affect *α-Man* expression in *rin* mutant. Interestingly, *α-Man* expression was induced when *rin* mutant was treated with ACC. However, as compared to the wild type (Ailsa craig), *α-Man* transcript induction level was less in *rin* mutant (**Figures 6B,D**). Therefore, ethylene may regulate *α-Man* expression in both RIN dependent and independent ways.

## Discussion

The physiological role of α-Man in ripening-associated fruit softening process has been demonstrated previously (Priya Sethu and Prabha, 1997; Suvarnalatha and Prabha, 1999; Hossain et al., 2009, 2010; Meli et al., 2010; Ghosh et al., 2011), but transcriptional regulation of *α-Man* during fruit ripening was not studied. To understand transcriptional regulation of *α-Man* during tomato fruit ripening, the promoter of *α-Man* was isolated from tomato and functionally characterized. *In silico* analysis revealed that the promoter region of *α-Man* contains several putative *cis*-acting elements, which may be involved in perceiving stimulus from different plant hormones and environmental signals during fruit ripening (**Supplementary Table S1**). The spatial and temporal expression patterns of the *GUS* reporter gene under the control of *α-Man* promoter have been determined through generating promoter::GUS transgenic tomato plants. In accordance to the fruit-ripening specific expression pattern of *α-Man* (**Figure 1B**; Meli et al., 2010), promoter activation during tomato ripening was also recorded (**Figures 2B,C**). Histochemical and fluorometric GUS assays, and transcript analysis in transgenic fruits suggested that the promoter is maximally active at the breaker stage of ripening (**Figures 2B,C**). As expected for fruit specific genes, *α-Man* promoter activity was not observed in whole seedling and in other parts (roots, stems, leaves, and flower) of transgenic plant (**Figure 3A**). However, sepals of flower showed very less GUS activity (**Figure 3A**). These results indicated that 1155 bp long promoter of *α-Man* used in this study was sufficient for driving fruit-ripening specific expression of *GUS* and contained all the *cis*-acting regulatory elements required for spatio-temporal regulation of the endogenous *α-Man*. These results substantiated the earlier findings suggesting the role of *α-Man* in ripening-associated fruit softening (Hossain et al., 2009, 2010; Meli et al., 2010; Ghosh et al., 2011).

In an effort to identify minimal *α-Man* promoter region required for expression in fruit, deletion analysis of the promoter was carried out (**Figures 4A,B**). Our data showed that GUS activity under the MD3 and MD4 promoter fragments was significantly less as compared to MD1 and MD2 truncated promoter and full length promoter (**Figure 4B**). These results suggested that the promoter region upstream to the MD3 fragment contained *cis*-acting element(s) required for the binding of the transcription factor involved in *α-Man* expression and full activity of the promoter. *In silico* analysis revealed that promoter region upstream to the MD3 fragment contains two CArG boxes at -413 and -387 bp (**Figure 4A**; **Supplementary Figure S4**) suggesting that *α-Man* expression could be regulated by the MADS box family transcription factor RIN. Although, MD3 and MD4 promoter fragments contain one CArG box at -62 bp that was not sufficient for the full activity of the *α-Man* promoter in fruit (**Figures 4A,C**). qRT-PCR analysis demonstrated that the expression of *α-Man* was down-regulated in *rin* mutant fruits (**Figure 5A**). Moreover, RIN also bound to the *α-Man* promoter in EMSA which suggested RIN-mediated direct transcriptional regulation of *α-Man* (**Figure 5B**). Moreover, *α-Man* promoter driven expression of GUS reporter was affected in *rin* mutant, further confirmed our hypothesis that RIN is involved in direct transcriptional regulation of the *α-Man* expression (**Figure 5C**). These results suggested that RIN positively regulates the transcriptional expression of *α-Man* during tomato ripening. RIN acts as a master regulator of fruit ripening by affecting expression of genes of various biological processes, both directly and indirectly (Ito et al., 2008; Fujisawa et al., 2011, 2012, 2013; Martel et al., 2011; Kumar et al., 2012; Qin et al., 2012). Our results demonstrate that RIN is a direct target transcriptional of *α-Man* during fruit ripening.

The role of ethylene in inducing tomato ripening is well established (Giovannoni, 2001). Therefore, to know the regulation of *α-Man* by ethylene, *α-Man* expression was analyzed by qRT-PCR after treatment with ACC, the precursor of ethylene. ACC was able to induce *α-Man* expression in wild type and promoter activation in MP::GUS transgenic seedlings (**Figures 6B,C**). These observations supported an essential role of ethylene in activating *α-Man* promoter during natural fruit ripening and early induction of *α-Man* at the breaker stage might be brought about by ethylene. Ethylene can regulate *α-Man* expression through ethylene receptors, e.g., ETR4 and NR and transcription factors such as ERF and ENI3 whose binding sites were identified in *α-Man* promoter. The role of ETR4 in the perception of ethylene has already been described (Kevany et al., 2008); however, its role in *α-Man* expression needs to be tested. The involvement of *NR* cannot be excluded as the expression of *α-Man* in *Nr* background was also suppressed (**Figure 6A**). Moreover, *in silico* analysis also revealed the presence of various *cis*-acting elements, related to the ethylene signaling, on the *α-Man* promoter (**Supplementary Table S1**). RIN is expressed prior to the onset of climacteric ethylene biosynthesis and *rin* mutant fruits show reduced ethylene production. Moreover, RIN regulates the expression of ethylene biosynthesis genes directly as well as indirectly (Ito et al., 2008; Fujisawa et al., 2011, 2013; Martel et al., 2011; Kumar et al., 2012; Qin et al., 2012; Zhong et al., 2013). RIN also known to regulate other ethylene signaling cascade genes including NR and ETR (Kevany et al., 2008; Martel et al., 2011; Kumar et al., 2012). Besides, the expression of RIN is also under the control of ethylene (Lincoln and Fischer, 1988; Fujisawa et al., 2013; Su et al., 2015). Interestingly, ethylene mediated induction of *α-Man* was less in *rin* mutant as compared to the wild type. Therefore, ethylene may regulate *α-Man* expression during fruit ripening through RIN as well as other ethylene regulated transcription factors such as EIN3 and ERFs. Taken together, these results suggest RIN-mediated direct transcriptional regulation of *α-Man* and ethylene may affect *α-Man* expression by RIN dependent and independent ways. In conclusion, the fruit ripening-specific promoter of *α-Man* has been identified, which could be a useful tool in fruit ripening related gene expression studies. The insights into transcriptional regulation of *α-Man* will also help us in understanding of molecular mechanism of fruit ripening regulation through RIN and ethylene. Further analyses of some ethylene related transcription factors and other regulators of ripening in context of α-Man and other *N*-glycan processing enzymes will clarify the regulation of *N*-glycan processing mechanism that triggers tomato fruit ripening.

## Author Contributions

MI, SG, VM, AD designed research; MI, SG, VM, AK, VK performed research; MI, SG, VM, NC, SC, AD analyzed data; MI, SG, AD wrote the paper. All authors read and approved the final manuscript.

## Conflict of Interest Statement

The authors declare that the research was conducted in the absence of any commercial or financial relationships that could be construed as a potential conflict of interest.

## Footnotes

1 http://solgenomics.net

2 https://sogo.dna.affrc.go.jp

3 www.genomatix.de
