# Supplementary material for: Fruit Ripening Regulation of α-Mannosidase Expression by the MADS Box Transcription Factor RIPENING INHIBITOR and Ethylene
Source: Front Plant Sci. 2016 Jan 21;7:10. doi: 10.3389/fpls.2016.00010 (PMC4720780; doi:10.3389/fpls.2016.00010)
Supplement: Supplementary file 1 [file Presentation_1.PDF]

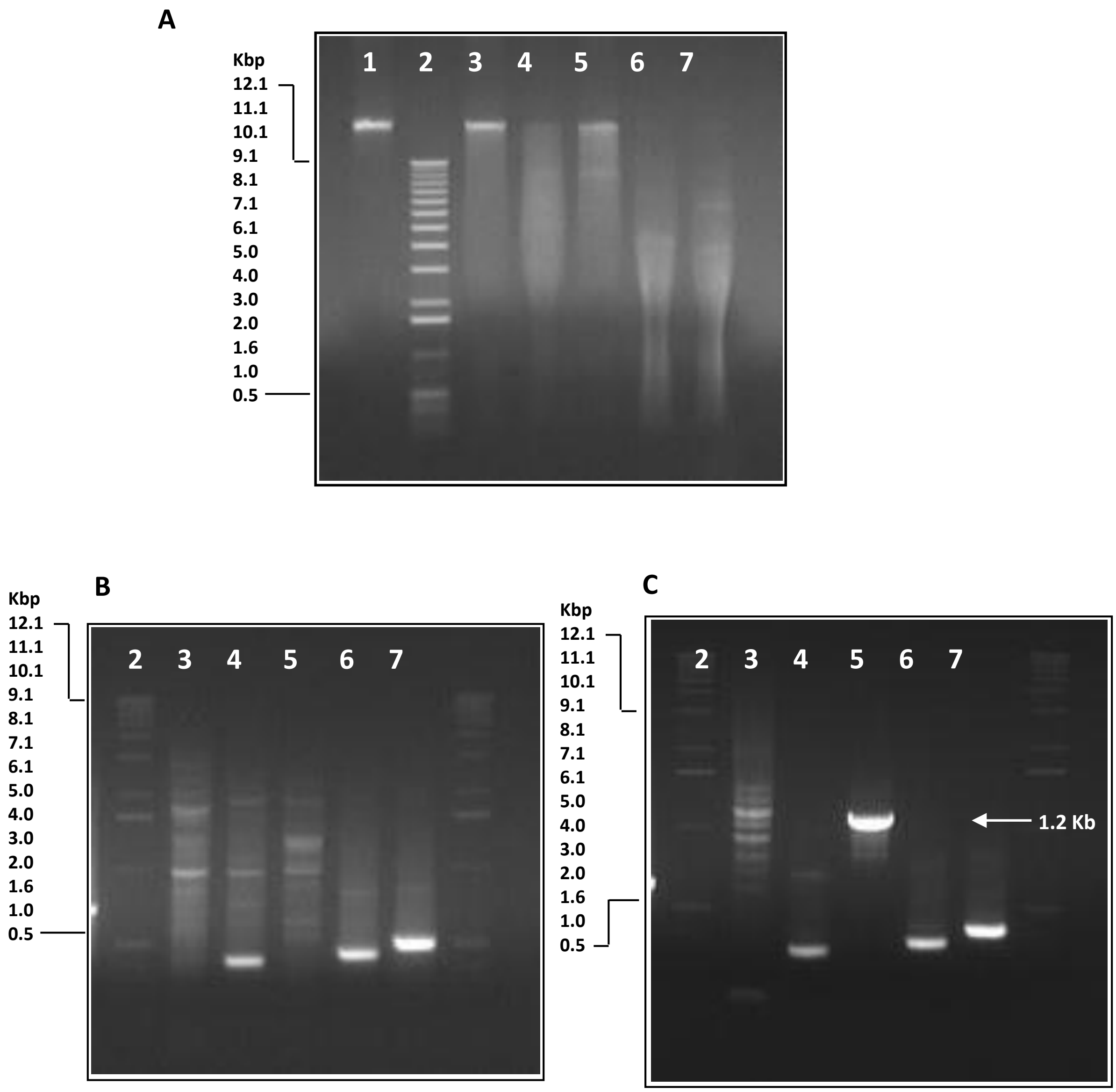

Figure S1: Promoter of *α-Man* was isolated by PCR based Genome walking method. (A) Genomic DNA was digested with *Pvu* II (lane 3), *Xmn* I (lane4), *Msc* I (lane 5), *Dra* I (lane 6) and *Ssp* I (lane 7). Adapters were ligated to the digested fragments and were referred as libraries. (B) Primary PCR with AP1 and GSP1 using the libraries constructed as mentioned in A. (C) Secondary PCR with the AP2 and GSP2 primers using diluted primary PCR product as the template. The arrow indicates the 1.2 kb fragment amplified from *Mse* I library, which was cloned in pGEM-T easy vector and sequenced. Lane 1 is undigested genomic DNA of tomato and lane 2 is DNA marker (Invitrogen, 1Kb ladder).

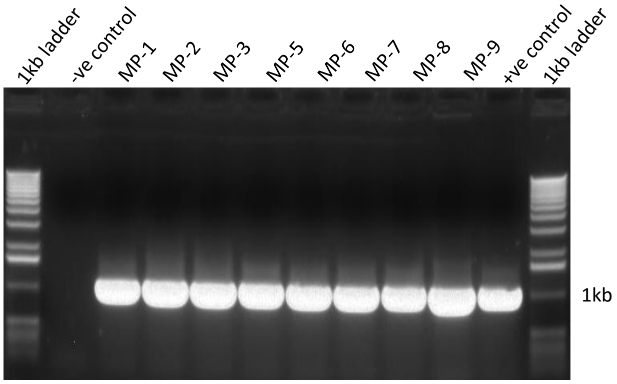

Figure S2: PCR confirmation of MP::GUS fusion transgenic (T_2) lines: Genomic DNA was isolated from leaves of MP::GUS fusion transgenic plants (T2). PCR amplification was done by using genomic DNA from MP::GUS fusion transgenic (T_2) lines and *α-Man* promoter specific forward and *GUS* specific reverse primer. –ve control: wild type/non-transformed plant, positive control: vector plasmid.

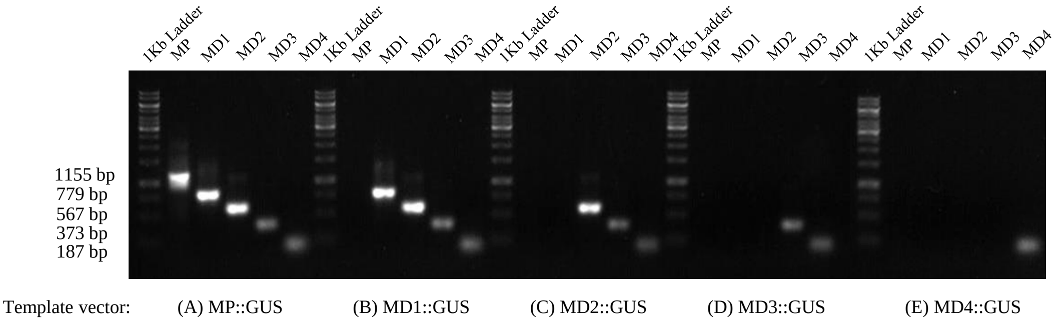

Figure S3: PCR confirmation of transformation of deletion constructs into *Agrobacterium tumifaciens*. PCR was done by using plasmid DNA of deletion constructs as template. MPF, MPD1, MPD2, MPD3, MPD4 were used as forward primers and MPR was used as reverse primer. (A) In the template MP::GUS, amplification of all the fragments was detected because of presence of full length promoter (1155bp). (B) In case of MD1::GUS, full length promoter (MP) was not amplified. (C) The bands of MP and MD1 were not appeared in MD2::GUS template. Similarly, no amplification of MP, MD1 and MD2 in MD3::GUS template (D) and MP, MD1, MD2, MD3 in MD3::GUS template was observed (E).

-1155
TATAATTAACTTCATTTTTTTTTTTTACCTAAAGTAAATTTATGTGTAATTTTATTATTATAATAAGTTCA

TATGTTTTTGTCGGAAATAATTTGAATTGCAGCAAATTTGTGAACTTAATAAAAGAATTTTTTCTGGAATT

ATCAGTACACAAAATTCAAATGGCATCCAACGCAAAAGATGCAACCTTTTCCATTTGCCCCATAAAAGTTA

GCAAAAATAAAGAAAGAAAGCAAGGGGAAAATAACAAAAGATGTTGAATACGACATAGTACTGACATCGGA

TAAAACCCTACTCGCTTTCTCCGTTTTAAAATAAGTGTTTATTTTATTTATTATAATAAAAAAATATCATC

MD1
TTAAATGTTTAACAAAAGAAT**A**CTCCAGAGCAATATAATAAAACTGACTAATCTATTAATAATTTTTAAT

CACGTTATAAAATTAAAAAAAAAGGAAATTTATAAAAATATACTATAATAAAAAAATATTTACCATTTATA

ACAATTACAATTTTTTCACTTCATCACTTTTAATTTATTTATAATACAAGTTTAATACATGTTATAAAGAT

MD2
AAATTTATTATTCAAATATAA**T**ATAAGTTTTAATGATGGATAATACATTTATCATACATTTTAATACACT

TATAATATAATGTGATAATTTTTTGCCAAACAAATAATATATATTTCAAAAACAATTATAATTCAAATATA

-422 -413 -396 -387
TTACATACATAATTTATTTCTAATA**CATATTAAAG**ATTTATCACAATATTA**CTATAAATGG**TAATAAATTA

MD3
AAAA**T**ATCGCTAAAATCAGTAATTATTTTTTAAAATGTAAATGTGATCTAAAATGGTGACGAATAGTTTC

CTAGTAGACGTGCTTTTTAACCTTATTTTTAACAATTATCAACACTTAGCTTTGTCAGCACCCAAGTCAAT

MD4
CCTAAGCAAAGAACCCATCATATAAAAATCATTAACACATTACTTATC**G**AAGACTTCACAAATCTTGATT

TTTGAATACGACCCTTTAGTATTAAGTTTTCCAATTAATCTTGTAGGTCCATTGGCTTTGATTCCCTTTCT

-71 -62
TGATTTATCTACAAAAATTTAGG**CTATTTATAG**AGATTTGGGGTTTATGGTTTAGTACATTCTCTTTCAGT

-1
GAACACTCTCAGCAATGGTGGAA

Figure S4: Sequence of *α-Man* promoter of tomato showing region selected for deletion mapping and position of CArG boxes.

Table S1. List of putative cis-acting regulatory elements identified within tomato *α-Man* promoter through *in-silico* analysis (NewPLACE, PlantCARE and MatInspector).

Cis-acting Elements	Position	Sequence
Ethylene insensitive 3 (EIN3) like factors, involved in ethylene regulated gene expression.	64-72 (-)	aTGAActta
	710-718 (-)	aTGTAtgta
	1034-1042 (-)	ATGGAccta
	62-71 (-)	CTATAAATAG
CArG Box	387-396 (-)	CCATTTATAG
	413-422 (-)	CTTTAATATG
W Box family	908-924 (-)	taggaTTGActtgggtg
Calcium regulated NAC-factors	911-931 (-)	ctttGCTTaggattgacttgg
	240-256 (-)	CatcttttGTTAttttc
	311-327 (-)	aataaacaCTTAtttta
MYB-like proteins	684-700 (+)	CaaaaacaATTAtaatt
	862-878 (-)	aaaataagGTTAaaaag
	975-994 (-)	aaAAATcaagatttgtgaa
Sucrose box	1051-1069 (-)	atAAATcaagaaagggaat
	771-786 (+)	ataaATTAaaaatat
Light response element	823-833 (+)	TgATCTaaaat
	1065-1076 (+)	TtATCTacaaa
	131-147 (-)	CtgataattccAGAAaa
Heat shock factors	1055-1072 (-)	TagataaatcaAGAAag
Dehydration responsive element binding factors	76-90 (-)	TatttCCGAcaaaaa
Ethylene-responsive element	679-687 (+)	ATTTCAAA
AuxRR-core (Element involved in auxin responsiveness)	1035-1041 (+)	GGTCCAT
TGACG-motif (Element involved in the MeJA-responsiveness)	835-839 (+)	TGACG
Element involved in circadian control	911-920 (+)	CAANNNNATC

Table S2. List of primers used in the study

Primer Sequence	Purpose
MPF: CCCAAGCTTATAATTAACTTCATTTTTTTT<br>MPR: GCTCTAGATTCCACCATTGCTGAGAGTGT	Preparation of tomato *α-Man* promoter::GUS fusion construct
RTTML: GTTGCTGCTTCAATACCACA<br>RTTMR: CTCCAAAGAGCTTCTAACCTG	qRT-PCR of *α-Man* in tomato
RTTAL: TTATCACCATTGGTGCTGAG<br>RTTAR: CGATGTTTCCATACAGATCCTT	qRT-PCR of tomato *actin* gene as endogenous control.
RTGUSF: CGGCAAAGTGTGGGTCAATA<br>RTGUSR: GCAATAACATACGGCGTGACA	qRT-PCR analysis of *GUS* gene
MPD1: CCCAAGCTTACTCCAGAGCAATATAATAAAACT<br>MPD2: CCCAAGCTTTATAAGTTTTAATGATGGATAATAC<br>MPD3: CCCAAGCTTTATCGCTAAAATCAGTAATTATT<br>MPD4: CCCAAGCTTGAAGACTTCACAAATCTTGATT<br>MPR: GCTCTAGATTCCACCATTGCTGAGAGTGT	Deletion mapping of *α-Man* promoter
